# Macroautophagy deficiency mediates age-dependent neurodegeneration through a phospho-tau pathway

**DOI:** 10.1186/1750-1326-7-48

**Published:** 2012-09-21

**Authors:** Keiichi Inoue, Joanne Rispoli, Hanoch Kaphzan, Eric Klann, Emily I Chen, Jongpil Kim, Masaaki Komatsu, Asa Abeliovich

**Affiliations:** 1 Departments of Pathology and Neurology, Taub Institute, Columbia University Medical Center, 650 W. 168th St., New York, NY, 10032, USA; 2 Center for Neural Science, New York University, 4 Washington Place, New York, NY, 10003, USA; 3 Department of Pharmacological Sciences and Stony Brook University Proteomics Center, Stony Brook University, Stony Brook, NY, 11794, USA; 4 Protein Metabolism Project, Tokyo Metropolitan Institute of Medical Science, Kamikitazawa 2-1-6, Setagaya-ku, Tokyo, 156-8506, Japan

## Abstract

**Background:**

Macroautophagy is an evolutionarily conserved mechanism for bulk intracellular degradation of proteins and organelles. Pathological studies have implicated macroautophagy defects in human neurodegenerative disorders of aging including Alzheimer’s disease and tauopathies. Neuronal deficiency of macroautophagy throughout mouse embryonic development results in neurodevelopmental defects and early postnatal mortality. However, the role of macroautophagy in mature CNS neurons, and the relationship with human disease neuropathology, remains unclear. Here we describe mice deficient in an essential macroautophagy component, Atg7, specifically within postnatal CNS neurons.

**Results:**

Postnatal forebrain-specific Atg7 conditional knockout (cKO) mice displayed age-dependent neurodegeneration and ubiquitin- and p62-positive inclusions. Phosphorylated tau was significantly accumulated in Atg7 cKO brains, but neurofibrillary tangles that typify end-stage human tauopathy were not apparent. A major tau kinase, glycogen synthase kinase 3β (GSK3β), was also accumulated in Atg7 cKO brains. Chronic pharmacological inhibition of tau phosphorylation, or genetic deletion of tau, significantly rescued Atg7-deficiency-mediated neurodegeneration, but did not suppress inclusion formation.

**Conclusions:**

These data elucidate a role for macroautophagy in the long-term survival and physiological function of adult CNS neurons. Neurodegeneration in the context of macroautophagy deficiency is mediated through a phospho-tau pathway.

## Background

The primary etiologies of neurodegenerative disorders, including Alzheimer’s disease (AD), frontotemporal dementia (FTD) and Parkinson’s disease (PD), remain largely unknown, but common pathological features suggest a role for altered protein degradation. For instance, proteinaceous intracellular inclusions composed in part of aggregated α-synuclein protein, termed Lewy bodies, typify PD brain pathology, whereas neurofibrillary tangles (NFT) and Pick bodies containing phosphorylated tau protein are commonly found in the context of taupathies such as AD and FTD. Rare, inherited familial forms of neurodegenerative diseases [1] are caused by mutations in genes encoding these accumulated proteins, such as α**-**synuclein [2,3] in PD and tau in FTD, but the vast majority of patients do not harbor known mutations. Thus, it has been hypothesized that in these ‘sporadic’ cases, pathological inclusions may reflect broadly defective protein degradation through mechanisms such as the ubiquitin-proteasome system (UPS) [4] and macroautophagy [5,6]. The latter is of particular interest because of its apparent role in the degradation of protein aggregates and inclusions [7].

Macroautophagy is a pathway of bulk cytoplasmic protein and organelle degradation characterized by double-membrane vesicles that engulf cargo and target it to lysosomes for degradation [8]. The pathway is typically induced in the context of starvation or other stressors. Defects in the macroautophagy process may theoretically occur at a variety of steps, from the initial formation of a pre-autophagosome limiting membrane, to the ultimate fusion of mature autophagosomes with the lysosomal compartment [9]. Macroautophagy defects have been well described on pathological analyses of brain sections from patients with a variety of neurodegenerative disorders, including AD, PD and FTD [5,10]. Furthermore, inherited genetic forms of neurodegeneration are associated with mutations in the macroautophagy-lysosomal pathway [11,12]. Finally, as macroautophagy dysfunction is a well-documented feature of aging, it has been implicated in the age-dependent nature of the major neurodegenerative disorders [5,9,10].

Genetically altered mice that are deficient in essential macroautophagy pathway components, Atg5 or Atg7, throughout neural development, display reduced neuronal survival and harbor ubiquitin-positive inclusions in the cell soma [13-16]. But surprisingly, prevention of inclusion formation in the context of Atg7-deficiency by a second genetic ablation of p62, which encodes an ubiquitin-binding protein associated with autophagosomes, does not suppress neurodegeneration, arguing against a toxic role for inclusions [17]. Thus, the mechanism of neuronal loss with macroautophagy deficiency, and how this relates to neurodegeneration, remains unclear.

Here we generated conditional Atg7-deficient mice specifically within mature CNS neurons. Atg7-deficient neurons were defective in the initiation of macroautophagy, and displayed a progressive degeneration with prominent inclusions that harbor ubiquitin, p62, phosphorylated tau and GSK3β. The mutant mice exhibited behavioral deficits consistent with the pathological changes. Furthermore, pharmacological or genetic suppression of tau phosphorylation effectively inhibited neurodegeneration in the context of Atg7-deficiency *in vivo*.

## Results

### Slowly progressive degeneration of postnatal **Atg7**-deficient hippocampal CA1 neurons

**Figure 1:**
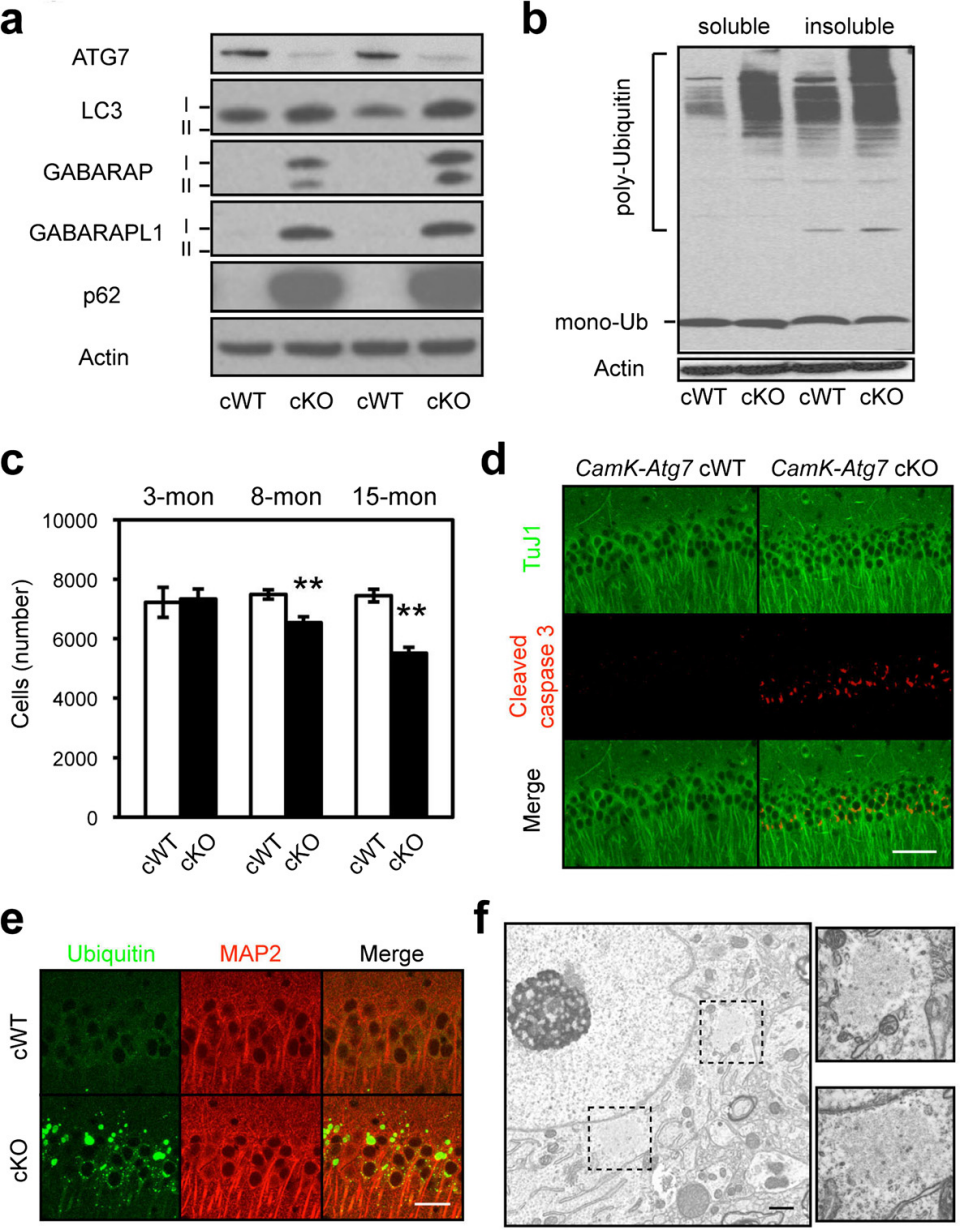
**Age-dependent neurodegeneration in forebrain-specific *Atg7*-deficient mice.** (**a**-**b**) Impaired macroautophagy in *CamK-Atg7* cKO mouse forebrain tissues including hippocampus and cortex. **a**, ATG7 protein was significantly reduced in *CamK-Atg7* cKO brains. Consistent with ATG7 change, mammalian Atg8 homologues (LC3, GABARAP and GABARAPL1) and macroautophagy substrate p62 were accumulated in *CamK-Atg7* cKO brains. **b**, Poly-ubiquitinated proteins were accumulated in both 0.5% TritonX-100-soluble and insoluble fractions of *CamK-Atg7* cKO forebrain. These results indicate that macroautophagy is impaired in the forebrains of *CamK-Atg7* cKO mice. (**c**-**d**) Slow progressive loss of hippocampal CA1 pyramidal neurons in *CamK-Atg7* cKO mice. **c**, Quantification of CA1 pyramidal neuron number. White bars, *CamK-Atg7* cWT. Black bars, *CamK-Atg7* cKO. n = 3 - 4 for each group. **, P < 0.01. **d**, Pyramidal neurons in the CA1 region of 8-month-old *CamK-Atg7* cKO mice were cleaved caspase-3-positive. Bar, 40 μm. (**e**) Cytoplasmic inclusions in 6-month-old *CamK-Atg7* cKO mice. Ubiquitin-positive (green) inclusions were present in MAP2-positive (red) hippocampal CA1 neurons of *CamK-Atg7* cKO mice, but were never seen in control *CamK-Atg7* cWT mice. Ubiquitin-positive inclusions were also positive for p62 (Additional file 1), as previously. Bar, 10 μm. (**f**) Electron microscopic analyses of cytoplasmic inclusions. The dashed squares outline two inclusions in the cytoplasm on neurons of *Atg7* cKO mice. At higher magnification, these inclusions display fibrillar and vesicular components and lack an outer membrane. Inclusions were never observed in control cWT mice. Bar, 500 nm.

Genetically altered mice that are deficient in an essential component of the macroautophagy machinery, Atg7 [18], specifically within mature forebrain neurons, were generated using a Cre-loxP strategy [19]. Briefly, we interbred mice that express bacterial Cre recombinase (CRE) under the control of the CamKII*α* gene regulatory sequences (*CamK-Cre*) [20] with *Atg7*^*flox/flox*^ mice [19]. CRE expression was limited to CA1 field pyramidal neurons of the hippocampus and glutamatergic neurons within the cerebral cortex [20], leading to ATG7 loss and prominent macroautophagy defects including the accumulations of LC3, GABARAP, GABARAPL1, and p62 in forebrain specific *Atg7* conditional knockout (*CamK-Atg7* cKO) mice (Figure 1a,b). Quantification of CA1 pyramidal neuron number revealed a significant reduction of approximately 25% in *CamK-Atg7* cKO mice at 1-year of age, while 3-month-old cKO mice maintained a normal complement of CA1 neurons (Figure 1c). Consistent with the neurodegenerative process, hippocampal CA1 neurons of 8-month-old *CamK-Atg7* cKO mice stained positively for cleaved caspase-3 (Figure 1d). In contrast, neither neuronal loss nor caspase-3 positive signal was observed in the cerebral cortex of 1-year-old *CamK-Atg7* cKO mice.

Furthermore, numerous ubiquitin-positive inclusions were apparent in essentially all Atg7-deficient CA1 cell bodies from 2-month of age, whereas these were never seen in the control *CamK-Atg7* cWT mice (Figure 1e). These inclusions were stained positive for p62 [17,21], which is a component of the macroautophagy machinery pathway (Additional file 1), and further confirmed the macroautophagy defect in forebrain neurons. In contrast, such inclusions were absent from the CA3 neurons (data not shown). Further analysis by electron microscopy revealed that these inclusions were composed of both filamentous and vesicular elements (Figure 1f).

We further compared *CamK-Atg7* cKO neurodegeneration with the effect of Atg7 deficiency in a second population of mature CNS neurons, midbrain dopamine (DA) neurons. To this end, we generated animals that express CRE under the control of the dopamine transporter (Dat) gene regulatory elements, and are homozygous for the floxed *Atg7* allele (*Dat*^*Cre/+*^*Atg7*^*flox/flox*^*; Dat-Atg7* cKO mice rather than *CamK-Atg7* cKO mice). *Dat-Atg7* cKO mice displayed a very similar pathological progression to *CamK-Atg7* cKO mice with cytoplasmic ubiquitin- and p62-positive inclusions, albeit the process is selective for midbrain DA neurons as expected (Additional file 2c,d). Neurodegeneration progresses appeared more rapid in the *Dat-Atg7* cKO mouse model than the *CamK-Atg7* cKO mouse model (25% midbrain DA neuron lost at 2-months of age and 38% lost at 4-month; Additional file 2a,b).

### *Atg7* deficiency in mouse postnatal forebrain neurons results in physiological and behavioral deficits

**Figure 2:**
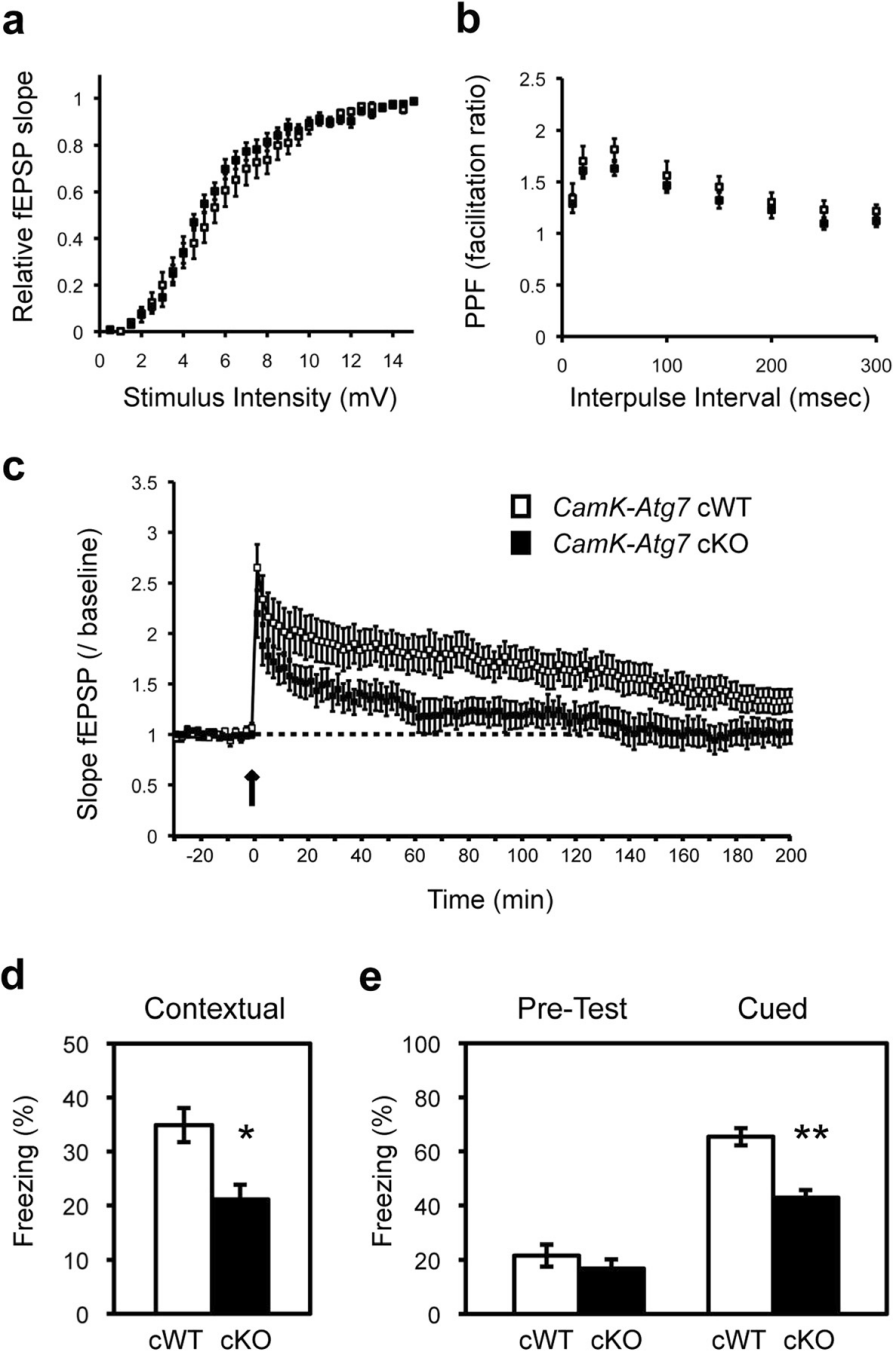
**Physiological and behavioral alterations in forebrain-specific *Atg7*-deficient mice.** (**a**-**c**) Forebrain-specific *Atg7* cKO mice display normal basal synaptic transmission but impaired LTP of the Schaffer collateral tract. Extracellular recording of field potentials was performed at area CA1 of acutely prepared hippocampal slices from 3-month-old male *CamK-Atg7* cKO mice and *CamK-Atg7* cWT littermates. **a**, Plots of fEPSP slope (normalized to maximal) versus stimulus intensity. There was no significant difference in baseline synaptic transmission between *CamK-Atg7* cKO mice and *CamK-Atg7* cWT littermates. n = 12 slices from 3 mice per genotype. **b**, Paired-pulse facilitation in *CamK-Atg7* cKO mice. The percent facilitation, determined by the ratio of the second fEPSP initial slope to the first fEPSP initial slope, is shown at interpulse intervals from 10 to 300 ms. n = 12 slices from 3 mice per genotype. **c**, Impaired LTP in *CamK-Atg7* cKO mice. Stable baseline responses were recorded prior to HFS (100 Hz HFS for 1 s) as indicated by arrow. One train of 100 Hz HFS elicited E-LTP in *CamK-Atg7* cKO mice that was decreased compared with that evoked in *CamK-Atg7* cWT mice. Open squares, *CamK-Atg7* cWT; Filled squares, *CamK-Atg7* cKO. n = 12 slices from 3 mice per genotype. p < 0.01 by repeated measures ANOVA. (**d**-**e**) Impaired fear memory of forebrain-specific *Atg7* cKO mice. **d**, *CamK-Atg7* cKO mice showed significant impairment in contextual fear memory 24 h after the training. **e**, *CamK-Atg7* cKO mice had impaired cued fear memory in the third day. Freezing level before the cue (tone) was not altered in *CamK-Atg7* cKO mice relative to *CamK-Atg7* cWT littermates (‘Pre-Test’). White bars, *CamK-Atg7* cWT (n = 10); Black bars, *CamK-Atg7* cKO (n = 8). *, P < 0.05. **, P < 0.01.

We further examined the physiological and behavioral consequences of Atg7-deficiency within forebrain neurons. Extracellular recording of field potentials were performed at Schaffer collateral synapses in area CA1 of acutely prepared hippocampal slices from 3-month-old male *CamK-Atg7* cKO mice and control *CamK-Atg7* cWT littermates. *CamK-Atg7* cKO mice showed normal input/output amplitudes in response to single stimuli (Figure 2a), as well as intact paired-pulse facilitation (PPF) at a variety of interpulse intervals (Figure 2b). These findings suggest that there are no gross differences in synaptic organization or baseline synaptic transmission in the cKO mice at this age. In contrast, early long-term potentiation (E-LTP) induced by a single high-frequency tetanic stimulation - a long-lasting protein synthesis-independent form of synaptic potentiation - was impaired in *CamK-Atg7* cKO slices (Figure 2c). In contrast, we note that long-term depression was intact in the cKO mice (data not shown). The relatively selective physiological impairment is unlikely to be secondary to the limited cell loss.

Next, we assessed forebrain-dependent fear conditioning in *CamK-Atg7* cKO mice and *CamK-Atg7* cWT mice. *CamK-Atg7* cKO mice did not show any increase in the ratio of freezing at their basal level. However, *CamK-Atg7* cKO mice showed a significant impairment in contextual fear conditioning relative to control *CamK-Atg7* cWT animals (Figure 2d). Furthermore, the cKO mice showed significant reduced freezing ratio in cued fear conditioning, whereas the basal freezing (‘Pre-Test’) was not changed (Figure 2e). Taken together, these data demonstrate forebrain physiological dysfunction, consistent with the selective forebrain pathology of *CamK-Atg7* cKO mice.

### Phospho-tau-positive inclusions in *Atg7*-deficient neurons

**Figure 3:**
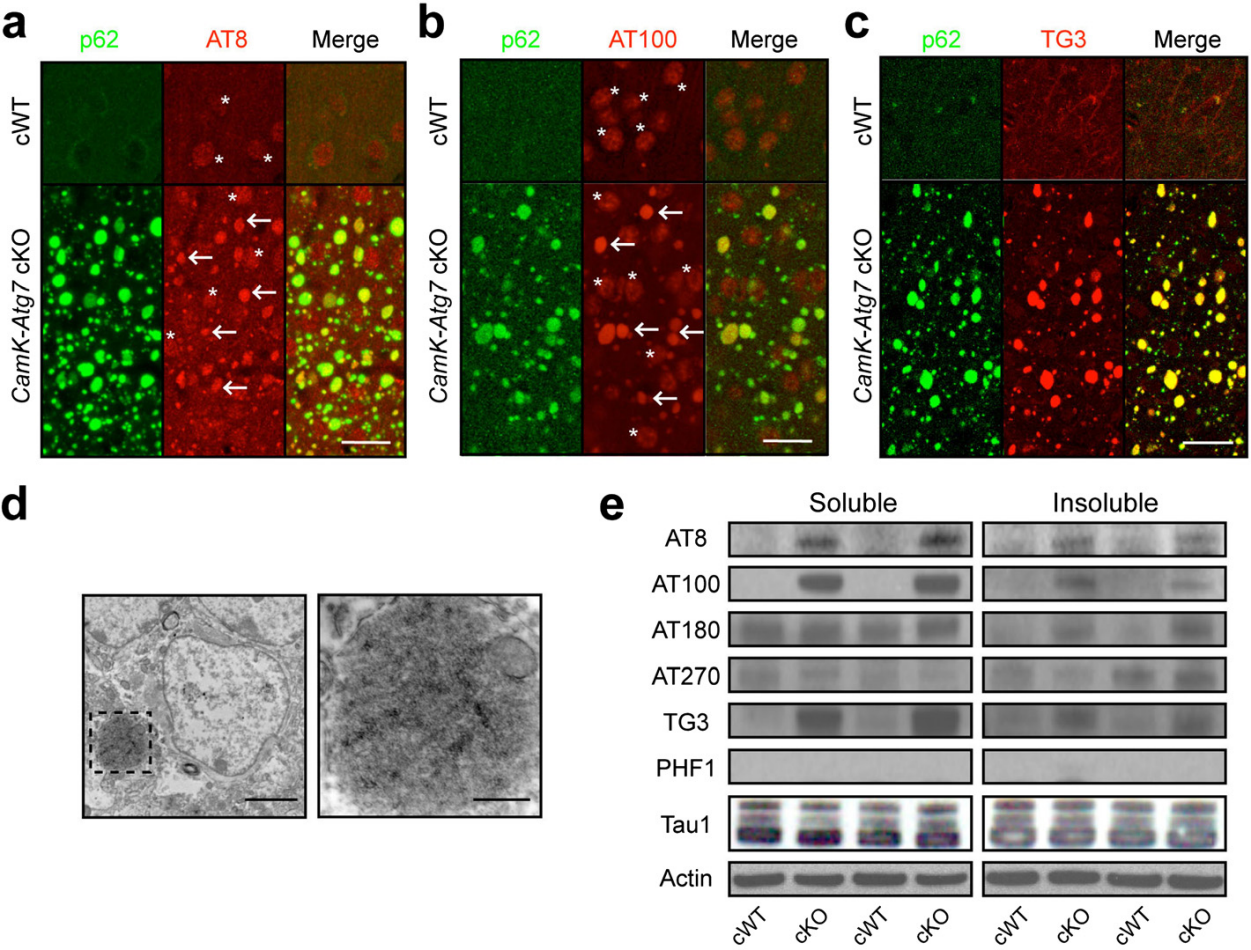
**Phospho-tau-positive inclusions in conditional *Atg7*-deficient mice.** (**a**-**c**) Phospho-tau-positive inclusions in cortical neurons of *CamK-Atg7* cKO mice. **a**, AT8, an antibody against phospho-tau at Ser202/Thr205 residues (red, arrows), stained p62-positive inclusions (green) in cortical neurons, with slight background signals (asterisks) in cell nucleus, of *CamK-Atg7* cKO mice. Bar, 10 μm. **b**, AT100, an antibody against phospho-tau at Ser212/Thr214 residues (red, arrows), stained p62-positive inclusions (green) in cortical neurons, with slight background signals (asterisks) in cell nucleus, of *CamK-Atg7* cKO mice. Bar, 10 μm. **c**, TG3, an antibody against phospho-tau at Thr231/Ser235 residues (red, arrows), stained p62-positive inclusions (green) in cortical neurons of *CamK-Atg7* cKO mice. Bar, 10 μm. (**d**) Immunoelectron microscopic analysis of phospho-tau-positive inclusion in *CamK-Atg7* cKO mice. Immunoelectron microscopic analysis of inclusions using the phospho-tau-specific antibody TG3 and horseradish peroxidase staining (dark speckles; contrast to Figure. 1f). At right is a high magnification image of the inset (dashed square in left). Bars, 2 μm (left) and 500 nm (right). (**e**) Phospho-tau levels are elevated in forebrain extracts of *CamK-Atg7* cKO mouse. Western blotting reveals that AT8-, AT100- or TG3-positive phospho-tau is significantly increased in both 0.5% Triton X-100-soluble and -insoluble fractions of *CamK-Atg7* cKO brain tissue extracts. AT270- or PHF1-positive phospho-tau and total tau [Tau1] were not changed. n = 5. Two independent samples are presented for each genotype, as labelled at bottom.

We investigated whether neurodegeneration caused by Atg7-deficiency is associated with typical pathological hallmarks of human neurodegenerative syndromes. Macroautophagy has previously been implicated in the clearance of various proteins implicated in human neurodegenerative syndromes including Alzheimer precursor protein (APP), α-synuclein, TDP-43, tau, and huntingtin [22-29]. However, direct *in vivo* evidence of an essential role for macroautophagy in the degradation of these proteins in forebrain is lacking. No accumulation of APP (or the APP-derived peptide fragmant β-amyloid), α-synuclein, or TDP-43 was detected in *CamK-Atg7* cKO mouse brain (Additional file 3a, b). However, cytoplasmic inclusions in Atg7-deficient CA1 pyramidal neurons and cerebral cortex neurons were prominently stained with multiple well-characterized antibodies to phospho-tau including AT8 (epitope at Ser202/Thr205), AT100 (epitope at Ser212/Thr214), and TG3 (epitope at Thr231/Ser235) [30,31] (Figure 3a-c). Similarly, electron microscopic analysis confirmed TG3-positive staining in the cytoplasmic inclusions of Atg7-deficient neurons (Figure 3d). We note that the inclusions were not stained with other antibodies for mature phospho-tau positive inclusions in human pathology, AT270 (epitope at Ser181) and PHF1 (epitope at Ser396/Ser404). Furthermore, the cytoplasmic inclusions did not stain with Thioflavin S, which marks mature NFTs in human tauopathies (Additional file 3c).

**Figure 4:**
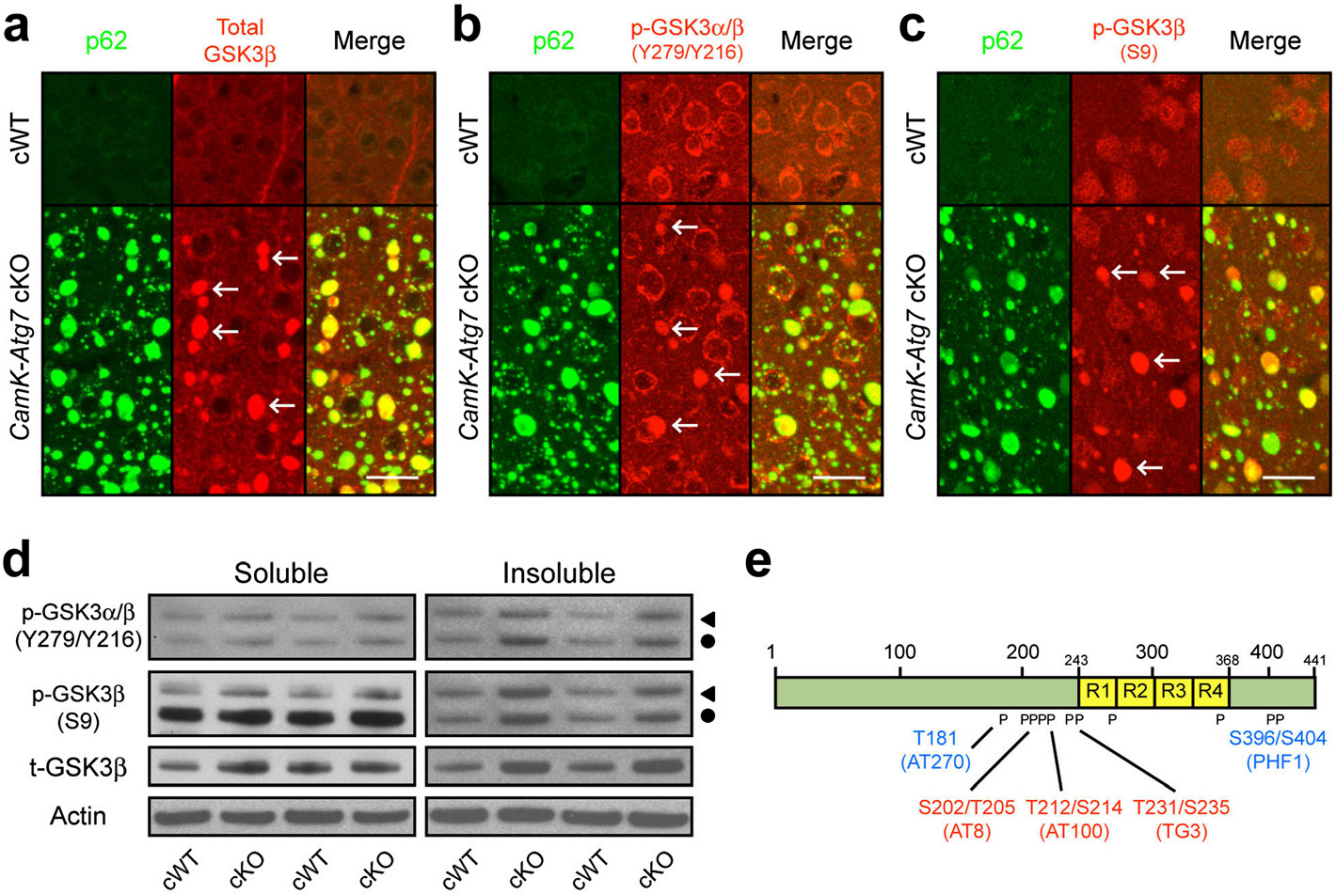
**GSK3β-positive inclusions in conditional *Atg7*-deficient mice.** (**a**-**c**) GSK3β-positive inclusions in cortical neurons of *CamK-Atg7* cKO mice. **a**, An antibody recognizing total GSK3β (red, arrows), stained p62-positive inclusions (green) in cortical neurons of *CamK-Atg7* cKO mice. Bar, 10 μm. **b**, Antibodies recognizing phosphorylated, activated form of GSK3β (Tyr279/Tyr216; in red, arrows) stained p62-positive (green) inclusions in cortical neurons of *CamK-Atg7* cKO mice. Bar, 10 μm. **c**, An antibody recognizing phosphorylated inactivated forms of GSK3β at Ser9 residues (red, arrows), stained p62-positive inclusions (green) in cortical neurons of *CamK-Atg7* cKO mice. Bar, 10 μm. (**d**) GSK3β levels are elevated in the forebrain extracts of *CamK-Atg7* cKO mouse. Western blotting reveals that phosphorylated forms of GSK3β (both activated Tyr216 residue and inactivated Ser9 residue) as well as total GSK3β were significantly increased in *CamK-Atg7* cKO brain tissue extracts. Triangle, GSK3α. Circle, GSK3β. n = 5 per group. (**e**) Summary of phosphorylation and antibody recognition sites of human tau protein. The phosphorylated sites (Ser202/Thr205 [AT8], Thr212/Ser214 [AT100], and Thr231/Ser235 [TG3] residues) in *Atg7*-deficient mice are shown in red. Non-phosphorylated sites are shown in blue. Phosphorylation sites are numbered according to the human tau protein by convention; the homologous corresponding phosphorylation sites in the mouse tau protein are each positioned 11 amino acids towards the amino terminus.

Quantitative Western blotting of forebrain extracts revealed that phospho-tau protein epitopes were broadly increased in forebrain tissues from *CamK-Atg7* cKO mice, whereas total tau protein appeared unaltered (Figure 3e). Several epitopes, including AT8, AT100, and TG3, were increased in both 0.5% TritinX-100-soluble and insoluble brain extracts (relative to *CamK-Atg7* cWT controls; Figure 3e), whereas AT180 accumulated only in insoluble extracts, and accumulation was not altered for AT270 and PHF1 (Figure 3e). The phospho-tau epitope staining pattern appeared very similar in midbrain DA neurons of *Dat-Atg7* cKO mice (Additional file 2e, Figure 4e). A similar phospho-tau pattern has previously been suggested to represent an early ‘pre-tangle’ state [32]; this may reflect an early stage of non-fibrillar tau aggregation prior to its assembly into paired helical filaments (PHF). Taken together, these data implicate phospho-tau accumulation in Atg7-deficiency-mediated neurodegeneration. However, the phospho-tau aggregates in the context of Atg7-deficient neurons do not replicate aspects of mature human tauopathy pathology.

### GSK3β staining at phospho-tau inclusions in *Atg7*-deficient neurons

Given the accumulation of phosphorylated -- but not total -- tau in Atg7-deficient neurons (Figure 4e), we hypothesized that a kinase that is known to phosphorylate tau, such as GSK3β, may be altered. Immunostaining of cortical neurons revealed dramatic re-localization of GSK3β, including both active (epitope at Tyr216) and inactive (epitope at Ser9) phosphorylated forms, to phospho-tau-positive and ubiquitin/p62-positive inclusions in Atg7-deficient neurons (Figure 4a-c). Western blot analysis confirmed that total and phosphorylated forms of GSK3α/β were increased in forebrain tissue extracts from *CamK-Atg7* cKO mice, compared to *CamK-Atg7* cWT mice (Figure 4d). Another kinase implicated in phosphorylation of tau, CDK5, did not appear to be re-localized to the inclusions in Atg7-deficient neurons [33] (Additional file 4d). Inclusions in Atg7-deficient neurons stained positively for a second microtubule-associated GSK3β substrate, phospho-CRMP2 [34] (Additional file 4a,b). In contrast, β-Catenin, a well-described GSK3β substrate in the context of Wnt signaling pathway, did not appear altered in staining in Atg7-deficient neurons (Additional file 4c). Thus, accumulated GSK3β in the context of Atg7-deficiency appears to display substrate specificity, perhaps related to subcellular re-localization at inclusions.

### Pharmacological or genetic inhibition of phospho-tau accumulation can rescue neuronal cell death *in vivo*

To examine the causality between phospho-tau and neurodegeneration in the context of Atg7-deficiency, we sought to determine whether neurons deficient in Atg7 could be effectively protected *in vivo* through the modulation of phospho-tau production. We focused these ‘rescue’ studies on *Dat-Atg7* cKO mice (rather than *CamK-Atg7* cKO mice) because the neurodegeneration progresses more rapidly in *Dat-Atg7* cKO mouse model than *CamK-Atg7* cKO mouse model, as noted above, and the degenerative and pathological processes are restricted to a single cell type in the *Dat-Atg7* cKO mice (midbrain DA neurons; Additional file 2a,b). *Dat-Atg7* cKO mice also displayed a very similar pathological progression to *CamK-Atg7* cKO mice with cytoplasmic ubiquitin- and p62-positive inclusions (Additional file 2c,d) that further stain for phospho-tau and GSK3β (Additional file 2e,f). Thus, analysis of pathology in *Dat-Atg7* cKO mice affords a more facile and accurate quantification of the cell autonomous impact of macroautophagy on the loss of mature CNS neurons.

**Figure 5:**
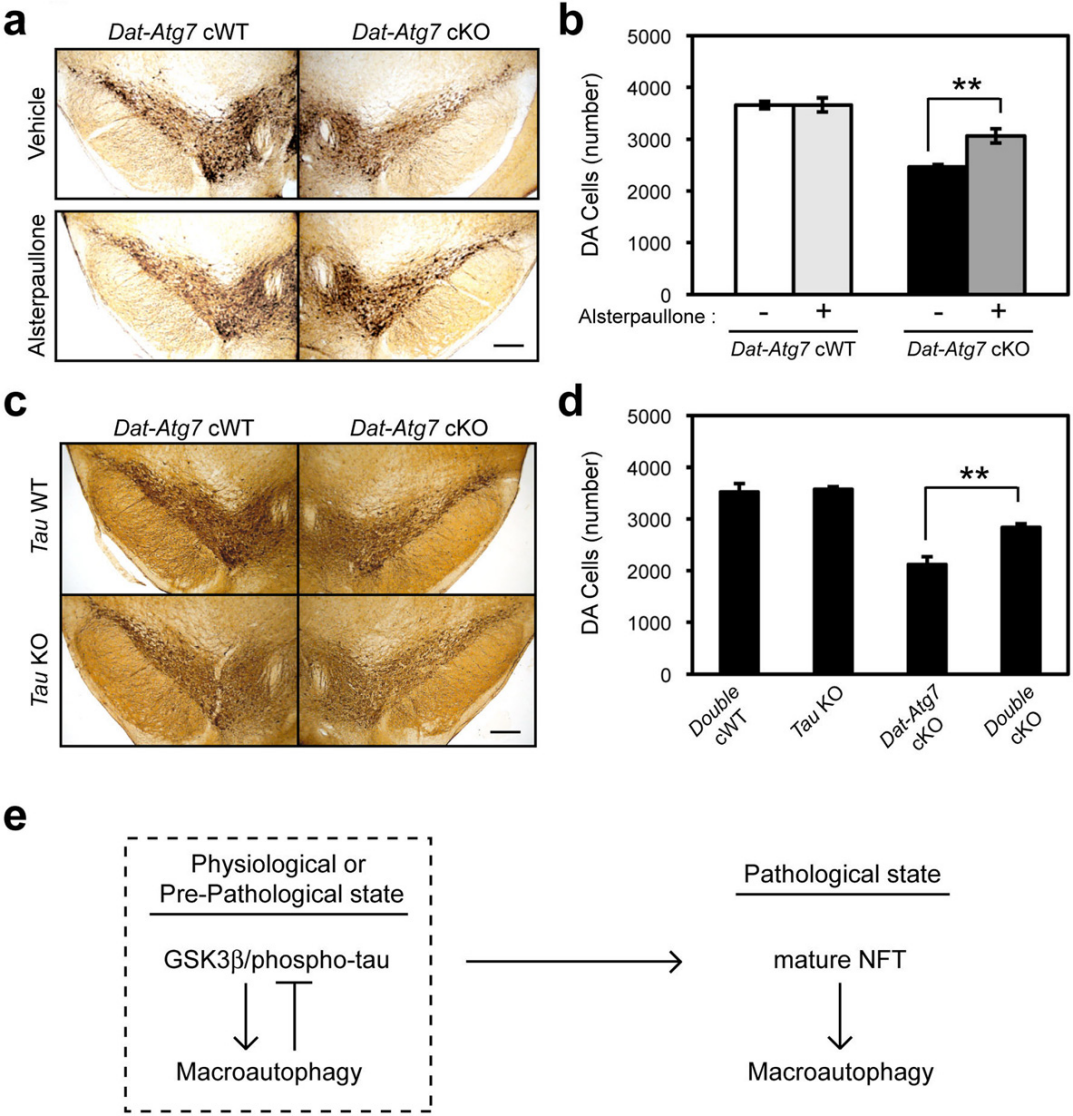
**Neuroprotection of *Atg7*-deficient CNS neurons *in vivo*.** (**a**-**b**) Pharmacological rescue of Atg7-deficient midbrain DA neuron loss by systemic injection of the selective GSK3β/CDK5 inhibitor Alsterpaullone. Five-week-old mice were dosed daily with 5 mg/kg Alsterpaullone by intraperitoneal injection for a 3-week period prior to analysis. TH-positive DA neuron loss was suppressed in Alsterpaullone-treated *Dat-Atg7* cKO mice, whereas Alsterpaullone-treated control *Dat-Atg7* cWT mice were unaffected. **b**, Quantification of TH-positive DA neuron number. **, P < 0.01. n = 5 - 6 / group. (**c**-**d**) Genetic rescue of DA neuron death in Atg7-deficient mice by secondary tau deletion. Immunostaining for TH-positive DA neurons at 3-month of age revealed significant protection of midbrain DA neurons from neurodegeneration in *Atg7* single KO mice (*Dat-Atg7* cKO) relative to controls (*Dat-Atg7/tau* double cWT). **d**, Quantification of TH-positive DA neuron number. **, P < 0.01. n = 4 - 6 / group. (**e**) Proposed model of phospho-tau and GSK3β regulation by macroautophagy. In physiological or pre-pathological states, basal macroautophagy regulates endogenous levels of phospho-tau and GSK3β (dashed square). In pathological states or with aging, macroautophagy is impeded, and phospho-tau and GSK3β are accumulated. These in turn lead to feedback induction of the macroautophagy pathway, although such feedback is ineffective in late-stage disease or in knockout mice.

To investigate the role of phospho-tau accumulation in Atg7-deficiency-induced neurodegeneration, *Dat-Atg7* cKO or *Dat-Atg7* cWT mice were treated chronically with a potent GSK3β/CDK5 inhibitor, Alsterpaullone (5 mg/kg/d, *i.p.*) for a period of 3 weeks starting at 5-week of age [35]. Alsterpaullone can inhibit the activities of GSK3β, as well as several other tau kinases (CDK1/2/5, GSK3α, and, to lesser extent, ERK1/2 and PKA) to suppress tau phosphorylation (Additional file 5a) [36]. At the end of the treatment course (8-weeks of age), pathological examination of the mice revealed that Alsterpaullone treatment led to a significant increase in the survival of midbrain DA neurons in *Dat-Atg7* cKO mice (24.3% increased survival, p < 0.01), whereas Alsterpaullone-treated control *Dat-Atg7* cWT mice appeared unaltered (Figure 5a, b). In contrast, ubiquitin-positive inclusions were unchanged in size and number in Alsterpaullone-treated *Dat-Atg7* cKO mice, whereas no inclusions were seen in Alsterpaullone-treated *Dat-Atg7* cWT mice (Additional file 5b, c). This is consistent with the previous report that the inclusion formation and neurodegeneration are independent in the context of macroautophagy deficiency [17]. These *in vivo* results are suggesting a protective effect by phospho-tau inhibition in the context of macroautophagy deficiency-induced neurodegeneration. As Alsterpaullone does display some inhibitory activity at kinases in addition to GSK3β, such as CDK5 [36], we cannot exclude additional *in vivo* kinase targets. But we note that unlike GSK3β, CDK5 did not appear modified or re-localized in *Dat-Atg7* cKO neurons (Additional file 4d).

Next, we examined the effect of tau-deficiency [37] in *Dat-Atg7* cKO mice. We generated *Dat-Atg7/tau* double cKO (*Dat*^*Cre/+*^*Atg7*^*flox/flox*^*tau*^*-/-*^) mice, and compared the loss of midbrain DA neuron in *Dat-Atg7* single cKO (*Dat*^*Cre/+*^*Atg7*^*flox/flox*^*tau*^*+/+*^ or *Dat*^*Cre/+*^*Atg7*^*flox/flox*^*tau*^*+/-*^) and *Dat-Atg7/tau* double cKO mice. The loss of midbrain DA neurons in *Dat-Atg7* cKO mice was significantly rescued in *Dat-Atg7/tau* double cKO mice at the age of 3-month (Figure 5c,d). Again, the formation of ubiquitin-positive inclusion was not changed in *Dat-Atg7/tau* double cKO mice (Additional file 5d,e). Consistent with the previous report that tau-deficiency alone led to no abnormality in the brain [37,38], neither neurodegeneration nor ubiquitin/p62-positive inclusions was seen in the midbrain DA neurons of *tau* KO mice (Figure 5c,d and Additional file 5d,e). Taken together, these approaches support a model whereby accumulation of phospho-tau contributes to neurodegeneration in the context of macroautophagy-deficiency, whereas the formation of ubiquitin/p62-positive inclusions is independent of phospho-tau signaling.

## Discussion

Here we investigated mechanisms of neurodegeneration downstream of Atg7-deficiency, and describe the pathological accumulation of GSKβ and phospho-tau proteins. A striking feature of neuropathology in the context of Atg7-deficiency is the redistribution of GSK3β to inclusions. We note that both GSK3β and phospho-tau are reported to be found in inclusions in tauopathy patient brain [39-43]. However, it is important to emphasize that Atg7-deficiency does not appear to induce a full tauopathy pathology, as not all phospho-tau epitopes are observed (e.g., PHF1 antibody is negative, Figure 4e), and amyloid staining with Thioflavin S, as well as electron microscopic analysis, do not support the presence of mature NFTs. A similar phospho-tau pattern has previously been suggested to represent an early ‘pre-tangle’ pathological state [32], thought to reflect non-fibrillar tau aggregation prior to assembly into PHFs. Such non-fibrillar hyperphosphorylated tau, rather than mature NFTs, may be the relevant toxic form *in vivo* in the context of neurodegeneration and behavioral impairment [44]. Hoozemans *et al.* reported phospho-tau-positive pre-tangles with accumulation of GSK3β, ubiquitin and p62 in postmortem specimens of AD patients [45], reminiscent of pathology in *Atg7*-deficient neurons *in vivo*. Phospho-tau pathology as seen in Atg7-deficient animals may broadly relate to neuronal dysfunction in neurodegeneration, as macroautophagy deficiency and phospho-tau are commonly observed in a broad array of neurodegenerative disorders including AD, PD, tauopathy, huntington disease, amyotrophic lateral sclerosis, and Gaucher disease [6,46-49]. Although genetic mutations in *ATG7* have not been described in human disease, mutations within other components of the macroautophagy-lysosomal pathway underlie tauopathies [50], consistent with our observations in the mouse model.

The *in vivo* pharmacological and genetic ‘rescue’ studies herein suggest a role for phospho-tau accumulation in neurodegeneration downstream of Atg7-deficiency. In contrast, prior attempts to rescue macroautophagy-deficiency associated neurodegeneration by preventing the formation of aggregates, by generation of double-knockout mice deficient in Atg7 as well as p62, were unsuccessful [17], suggesting that inclusion formation *per se* is insufficient for degeneration. It is interesting to note that nonetheless, p62 deletion does rescue the Atg7 deficiency-associated cell loss in hepatocytes [17], and thus degenerative pathways downstream of macroautophagy loss appear cell type-specific. Furthermore, within the CNS, various neuronal subtypes appear to be differentially affected by macroautophagy deficiency. Purkinje neurons deficient in Atg7 display axonal swellings and are rapidly lost [51]. TH-positive midbrain DA neurons display axonal dystrophy and degeneration, ubiquitin/p62-positive inclusions, and delayed cell loss and locomotor dysfunction [52]. Although tau pathology was not investigated in these other models, staining for the Parkinson’s disease associated proteins α-synuclein and leucine rich repeat kinase-2 (LRRK2) was reported in Atg7-deficient DA neurons [52]. We failed to detect evidence of α-synuclein accumulation in our analysis of either midbrain DA neuron-selective or forebrain neuron-selective Atg7-deficient mice detailed above (data not shown). Such discrepancies may reflect differences in the selectivity or timing of the CRE-mediated deletion strains used in the different studies, or selective sensitivity to macroautophagy loss across distinct neuron types. We note that phospho-tau pathology was apparent in the context of either midbrain DA neuron-selective or forebrain neuron-selective Atg7-deficiency.

The molecular basis of GSK3β and phospho-tau accumulation in Atg7-deficient neurons remains to be elucidated. We cannot exclude the possibility that GSK3β accumulation is a secondary effect of phospho-tau accumulation. A recent study described intracellular redistribution of GSK3β to multivesicular bodies, albeit in the context of Wnt pathway modulation [53]. As multivesicular bodies directly associate with the macroautophagy machinery, it is possible that GSK3β degradation is selectively modified with macroautophagy loss [54]. Although GSK3β is a strong candidate for the relevant upstream kinase, we hypothesize the involvement of other kinase pathways, particularly given the multiple targets of the pharmacological kinase inhibitor used, Alsterpaullone. Furthermore, Alsterpaullone-mediated protection may be mediated through targets in addition to tau, which would be of further interest.

We propose a role for basal macroautophagy in regulating the metabolism of phospho-tau proteins at physiological or pre-pathological state (Figure 5e). In the context of macroautophagy loss, GSK3β and phospho-tau are accumulated, reminiscent of early pathology that precedes human tauopathy. It is interesting to note that both GSK3β and tau are believed to be potent upstream regulators of macroautophagy [55-58]. We hypothesize that this may reflect a feedback loop, where defective macroautophagy leads progressively to more accumulation of phospho-tau and GSK3β, and in turn the accumulated phospho-tau and GSK3β both induce macroautophagy activity. Initially such feedback may be effective, although the accumulated proteins form inclusions. But once macroautophagy deficiency is complete, as in late-stage disease or in knockout mice, this feedback would be ineffective. Thus, such a feedback circuit may be an important pathway to rejuvenate the macroautophagy pathway, which is known to wane with aging [59].

## Conclusions

*Atg7* cKO in mouse forebrain neurons led to an age-dependent neurodegeneration with ubiquitin/p62-positive and phospho-tau/GSK3β inclusions, but not the full pathological features of NFTs in tauopathy. Pharmacological or genetic inhibition of tau phosphorylation *in vivo* successfully rescued neurodegeneration in the context of macroautophagy-deficiency. As GSK3β and tau are also upstream inducers of macroautophagy, this implicates a negative feedback loop in human pathology.

## Methods

### Animal

*CamK-Cre* transgenic mice, *Dat*^*Cre/+*^ mice, *Atg7*^*flox/flox*^ mice, hAPP-Tg and *tau* KO mice, used in this study were generated previously [19,20,37,60-62]. *CamK-Cre* Tg and *tau* KO mice were purchased from Jackson Laboratories. All animals were maintained in the animal facility of the Columbia University Medical Center. Experimental protocols were approved by the Institutional Animal Care and Use Committee. Genomic DNA extracted from mouse tails was amplified by PCR for genotyping using standard methods. The PCR primers are the followings: 5’-AGA TGT TCG CGA TTA TC-3’, 5’-AGC TAC ACC AGA GAC GG-3’ for Cre transgene; 5’-TGC TCT GTG AAC TGC CCT GTT T-3’, 5’-TGT TCC TGT GCA CTG CCT CAT T-3’ for *Atg7* wild-type allele; 5’-CTT GGG TGG AGA GGC TAT TC-3’, 5’-AGG TGA GAT GAC AGG AGA TC-3’ for *Atg7* floxed allele.

### Histology

Mice were perfused and fixed in 4% paraformaldehyde and post-fixed at 4°C overnight, 50 μm coronal brain sections were generated using a vibratome. The sections were blocked with PBS containing 5% normal donkey serum [NDS], 0.2% Triton X-100 [Tx] for 1 h, and incubated with the solution (PBS, 1% NDS, 0.2% Tx) containing primary antibody at 4°C overnight. The following antibodies were used; anti-TH (P60101, Pel-Freez), anti-TuJ1 (MMS-435P, COVANCE), anti-MAP2 (AB5622, Millipore), anti-cleaved caspase-3 [Asp175] (#9661, Millipore), anti-active caspase-3 (AB3623, Cell Signaling Technology), anti-ubiquitin (Sigma-Aldrich), anti-p62 (03-GP62-C, American Research Products), anti-Aβ [4G8] (SIG39200, COVANCE), anti-Aβ [6E10] (SIG39300, COVANCE), anti-αSynuclein (610786, BD Bioscience) (AB5038, Millipore) (ab1903, ab24715, Abcam), anti-phosph-tau TG3 and PHF1 (gifts from Dr. Peter Davies, Alberts Einstein College of Medicine), anti-phospho-tau AT8, AT100, AT180, and AT270 (Pierce), anti-total GSK3β (#9315, Cell Signaling Technology), anti-phospho-GSK3α/β [Y279/Y216] (ab52188, Abcam), anti-phospho-GSK3β [S9] (ab30619, Abcam), anti-total CRMP2 (#9393, Cell Signaling Technology), anti-phospho-CRMP2 [T514] (#9397, Cell Signaling Technology), anti-Cdk5 (MAB5410, Millipore), anti-p35/25 (#2680, Cell Signaling Technology), anti-β-catenin (#9581, 9587, Cell Signaling Technology), and anti-β-catenin (#610154, BD Biosciecnes). For secondary detection, Cy3- or FITC-conjugated antibodies were incubated for 1 h (Jackson ImmunoResearch). Photographs were taken using a Zeiss LSM 510 Meta confocal microscope.

### Neuron counting

To obtain neuronal cell count, 50 μm coronal brain sections were made using a vibratome. In order to count CA1 neurons, the first 30 sections from the rostral hippocampus were stained with rabbit anti-MAP2 antibody (AB5622, Millipore) at a dilution of 1:500, as well as NeuroTrace^TM^ Fluorescent Nissl stain (N21480, Invitrogen). MAP2-positive neurons were visualized using a Cy3-conjugated secondary antibody (Jackson ImmunoResearch). MAP2 and Nissl double-positive neurons in the CA1 regions were counted manually. In order to count TH-positive neurons, sections covering the entire substantia nigra (25-30 sections / mouse) were stained with sheep anti-TH antibody (P60101, Pel-Freez) at a dilution of 1:250. TH-positive neurons were visualized using the ABC Kit (PK6106, Vector Laboratories) and DAB (SK4100, Vector Laboratories). TH-positive neurons in the substantia nigral regions were counted manually under the light microscope.

### Electron microscopy

Electron microscopic analysis was as described [61]. Anesthetized mice were perfused and fixed in PBS containing 4% paraformaldehyde and 0.5% gultaralaldehyde. The brains were post-fixed at 4°C for 2 h, and the 80 μm vibratome sections were made. The sections were treated in 1% osmium tetroxide, then dehydrated in pure ethanol and infiltrated overnight with Epon 812. Epon was polymerized at 60°C for 24 h, cooled and embedded in a larger Epon capsule. Ultrathin sections were cut with an MT5000 ultramicrotome, stained with uranyl acetate and lead citrate. Images were taken with a JOEL 100S Electron Microscope (JOEL USA).

### Tissue fractionation

Preparation of soluble and insoluble fractions was performed as described with some modifications [14]. Cortical and hippocampal tissues from mouse brains were homogenized in 5× volume of ice-cold 0.25M sucrose buffer (50mM Tris-HCl [pH7.6]) containing protease inhibitors (P8340, Sigma) and phosphatase inhibitors (#78420, Thermo Scientific). The homogenized tissues were centrifuged at 500× g for 10 min at 4°C. The supernatants were lysed with an equal volume of cold sucrose buffer containing 1% Triton X-100. The lysates were centrifuged at 13,000× g for 15 min at 4°C. The supernatants contained the soluble fraction. The pellets were resuspended in 1% SDS in PBS (insoluble fraction). Both fractions were subjected to standard Western Blotting analysis. The antibodies used here are: anti-phospho-tau AT8, AT100, AT180, AT270, TG3 and PHF1, anti-Tau1 and anti-Actin (ab3280, Abcam). Horseradish peroxidase-conjugated secondary antibodies (Jackson ImmunoResearch) and SuperSignal West Pico or Dura (#34077, 34075, Pierce) were used for detection.

### Electrophysiology

Brains from *CamK-Atg7* cWT and cKO mice littermates (~12 weeks of age) were quickly removed and transverse hippocampal slices (400 μm) were isolated with a Leica VT1200 Vibratome (Leica, Bannockburn, IL), and placed in ice-cold cutting solution (CS: 110 mM Sucrose, 60 mM NaCl, 3 mM KCl, 1.25 mM NaH_2_PO_4_, 28 mM NaHCO_3_, 0.5 mM CaCl_2_, 7 mM MgCl_2_, 5 mM Glucose, 0.6 mM Ascorbate. Slices were placed in an interface chamber (Scientific Systems Design, Mississauga, Ontario, Canada) and maintained at 32°C in ACSF (2 ml/min) containing 125 mM NaCl, 2.5 mM KCl, 1.25 mM NaH_2_PO_4_, 25 mM NaHCO_3_, 25 mM D-glucose, 2 mM CaCl_2_, and 1 mM MgCl_2_. All solutions were constantly caboxygenated with 95% O_2_ + 5% CO_2_. Slices were allowed to recover for 120 min on the electrophysiology rig prior to experimentation. Bipolar stimulating electrodes (92:8 Pt:Y) were placed at the border of area CA3 and area CA1 along the Schaffer-Collateral pathway. ACSF-filled glass recording electrodes (1–3 MΩ) were placed in stratum radiatum of area CA1. Basal synaptic transmission was assessed for each slice by applying gradually increasing stimuli (0.5–15V), using a stimulus isolator (A-M Systems, Carlsborg, WA) and determining the input:output relationship. All subsequent stimuli applied to slices was equivalent to the level necessary to evoke a fEPSP that was ~40% of the maximal initial slope that could be evoked. Synaptic efficacy was continuously monitored (0.05 Hz). Sweeps were averaged together every 2 min. fEPSPs were amplified (A-M Systems Model 1800) and digitized (Digidata 1440, Molecular Devices, Sunnyvale, CA) prior to analysis (pClamp, Molecular Devices, Sunnyvale, CA). Stable baseline synaptic transmission was established for 30 min. Slices were given high-frequency stimulation (HFS) to induce long-term potentiation (LTP) using one train of 100 Hz for one second. Stimulus intensity of the HFS was matched to the intensity used in the baseline recordings. fEPSP initial slopes from averaged traces were normalized to those recorded during baseline. Two-way RM-ANOVA were used for electrophysiological data analysis with p < 0.05 as significance criteria.

### Fear conditioning

10-13-mon-old male *CamK-Atg7* cWT or *CamK-Atg7* cKO mice were used (n = 8 - 10). The mice were placed in a conditioning chamber (Med Associates) for 2 min before the onset of a tone (conditioned stimulus) (30 s, 85 dB sound at 2800 Hz) and conditioned by a single electrical foot shock (0.45 mA) in the last 2 s. The mice were left in the chamber for another 30 s and placed back into their home cage. Contextual fear learning was measured in the same chamber 24 h after the training by monitoring the freezing for 5 min without electrical shock. Cued fear learning was measured 24 h after the contextual testing. The mice were placed in a novel chamber for 2 min (pre-conditioning). After that, the mice were exposed to the conditioned stimulus for 3 min, and the freezing was monitored. Freezing behavior was scored using FreezeView software (Med Associates Inc.).

### Drug injection

Five-week-old *Dat-Atg7* cWT and *Dat-Atg7* cKO mice were treated with Alsterpaullone (A1136, A.G. Scientific) [35]. The drug was dissolved in saline containing 20% DMSO/ 25% Tween80, sonicated, and injected intraperitoneally at a dose of 5 mg/kg every day for 3 weeks. After the final injection, the mice were perfused and processed for histological analyses. We used *Dat-Atg7* cWT mice as controls for *Dat-Atg7* cKO mice, to address potential phenotypes due to Cre transgene inserted at the DAT locus [62].

### Statistical analysis

All comparisons between groups were made using the Mann-Whitney U-test (for two samples) or non-repeated measures ANOVA (for multiple samples). The values are expressed as the means ± S.E. A *p* value less than 0.05 is considered significant.

## Competing interests

The authors declare no competing interests.

## Authors’ contributions

KI, JR, HK, EC, JK, and MK performed the experiments. KI, HK, EK, EC, and AA analyzed the results. KI and AA designed the study and wrote the manuscript. All authors read and approved the final manuscript.

## Supplementary Material

Additional file 1 **Intracellular ubiquitin and p62 positive inclusions in 6-month-old *CamK-Atg7* cKO mice.** Ubiquitin-positive inclusions are almost completely overlapped with p62-positive inclusions in the cerebral cortex of *CamK-Atg7* cKO mice. Ubiquitin/p62-positive inclusions were already seen at 2-month-old *Atg7* cKO mice. Bar, 10 μm. Click here for file

Additional file 2 **Progressive neurodegeneration in midbrain DA neuron-specific Atg7-deficient (*Dat-Atg7 *cKO) mice.** (a-b) Progressive loss of DA neurons in *Dat-Atg7* cKO mice. a, Representative midbrain sections stained with polyclonal antibody specific for TH.Bar, 250 μm. b, Quantification of TH-positive DA neuron number as in (a). White bars, *Dat-Atg7* cWT. Black bars, *Dat-Atg7* cKO. n = 3 –7 for each group. **, P<0.01. (c-d) Cytoplasmic and dendritic inclusions in *Dat-Atg7* cKO mice. Ubiquitin-positive (c, red) and p62/SQSTM1-positive (d, red) inclusions were present in TH-positive DA neurons (green) of 1-month-old *Dat-Atg7* cKO mice, but were never seen in control *Dat-Atg7* cWT mice. Bars, 10 μm. (e) Phospho-tau-positive inclusions in TH-positive DA neurons in *Dat-Atg7* cKO mice. Phospho-tau specific antibodies (red), AT8, AT100, and TG3, stained inclusions (arrows) in the soma and dendrites of TH-positive DA neurons (green) in *Dat-Atg7* cKO mice. AT8, tau phosphorylated at Ser202/Thr205. AT100, tau phosphorylated at Ser212/Thr214. TG3, tau phosphorylated at Thr231/Ser235. Bar, 10 μm. (f) GSK3β-positive inclusions in TH-positive DA neurons in *Dat-Atg7* cKO mice. Antibodies recognizing total, activated form (Tyr279/Tyr216), and inactivated form (Ser9) of GSK3β (red), stained the inclusions (arrows) in TH-positive DA neurons (green) in *Dat-Atg7* cKO mice. Bar, 10 μm. Click here for file

Additional file 3 **APP/Aβ-negative, α-Synuclein-negative, and Thioflavin S-negative inclusions in *CamK-Atg7 *cKO mice.** (a) The ubiquitin-positive inclusions (green) in 1-year-old *CamK-Atg7* cKO mice did not contain mouse Aβ (red) (left). 4G8, monoclonal antibody to amino acid residues 17-24 of Aβ, was used. Ten-month-old transgenic mice bearing a mutant form of human APP (K670N/M671L/V717F, J20 line) were used as positive control for Aβ plaque staining (right). Similar negative results were obtained by 6E10, another antibody to amino acid residues 1-16 of Aβ (data not shown). Bars, 10 μm. (b) The ubiquitin-positive inclusions (green) in 1-year-old *CamK-Atg7* cKO mice did not contain mouse α-Synuclein (red). Four different anti-α-synuclein antibodies were used for the double staining. None of four anti-α-synuclein antibodies could detect any positive signals (red) in ubiquitin-positive inclusions (green). Bar, 10 μm. (c) Ubiquitin-positive inclusions (red) in 1-year-old *CamK-Atg7* cKO mice were negative for Thioflavin S staining (green, left). Thioflavin S stains plaques from β-amyloid and neurofibrillary tangles. Ten-month-old J20 APP transgenic mice were used as positive control for Thioflavin S staining (right). Bars, 20 μm. Click here for file

Additional file 4 **Immunohistochemical analyses of Atg7-deficient neurons.** (a-b) CRMP2-positive inclusions in cortical neurons in *CamK-Atg7* cKO mice. a, An antibody recognizing total CRMP2 (red), stained p62-positive inclusions (green) in cortical neurons of *CamK-Atg7* cKO mice. Bar, 10 μm. b, An antibody recognizing phosphorylated forms of CRMP2 at Thr514 residues (red), stained p62-positive inclusions (green) in cortical neurons of *CamK-Atg7* cKO mice. Bar, 10 μm. (c) β-Catenin-negative inclusions in TH-positive DA neurons in *Dat-Atg7* cKO mice. Antibodies recognizing β-Catenin (red) did not stain the inclusions in TH-positive DA neurons (green) in *Dat-Atg7* cKO mice. Bar, 10 μm. (d) CDK5-negative inclusions in TH-positive DA neurons in *Dat-Atg7* cKO mice. Antibodies recognizing CDK5 (red) did not stain the inclusions in TH-positive DA neurons (green) in *Dat-Atg7* cKO mice. Bar, 10 μm. Click here for file

Additional file 5 **Neuroprotection of Atg7-deficient CNS neurons *in vivo.*** (a) Alsterpaullone can reduce phospho-tau accumulation in the context of macroautophagy inhibition. N2a cells were treated with 1 μM Alsterpaullone in the context of 100 μM chloroquine treatment for 24 h. Cells were lysed in RIPA buffer and subjected to standard Western blotting analysis. Phospho-tau levels were detected by AT8 antibody. (b-c) Ubiquitin-positive inclusion formation was unaffected by systemic injection of Alsterpaullone in the context of *Dat-Atg7* cKO mice. Bar, 10 μm. c, Quantification of ubiquitin-positive inclusion number per TH-neuron in *Dat-Atg7* cKO mice. No inclusions were observed in *Dat-Atg7* cWT mice. n > 60 neurons per genotype. (d-e) Ubiquitin-positive inclusion formation (red) was not changed in TH-positive DA neurons (green) of *Dat-Atg7/tau* double cKO mice relative to *Dat-Atg7* cKO mice. No ubiquitin-positive inclusions were detected in *tau* KO mice. Bar, 10 μm. e, Quantification of ubiquitin-positive inclusion number per TH-neuron in *Dat-Atg7/tau* double cKO mice. n > 60 neurons per genotype. Click here for file
