# Supplementary figures and images for: Macroautophagy deficiency mediates age-dependent neurodegeneration through a phospho-tau pathway
Source: Mol Neurodegener. 2012 Sep 21;7:48. doi: 10.1186/1750-1326-7-48 (PMC3544596; doi:10.1186/1750-1326-7-48)

# Additional File 1

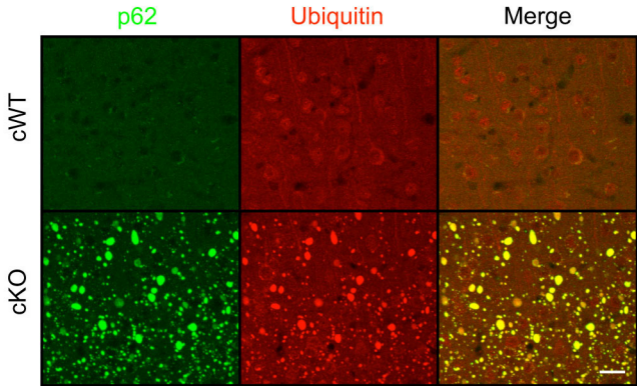

Supplement: Additional file 1 — Intracellular ubiquitin and p62 positive inclusions in 6-month-old CamK-Atg7 cKO mice. Ubiquitin-positive inclusions are almost completely overlapped with p62-positive inclusions in the cerebral cortex of CamK-Atg7 cKO mice. Ubiquitin/p62-positive inclusions were already seen at 2-month-old Atg7 cKO mice. Bar, 10 μm. [file 1750-1326-7-48-S1.pdf]

# Additional File 2

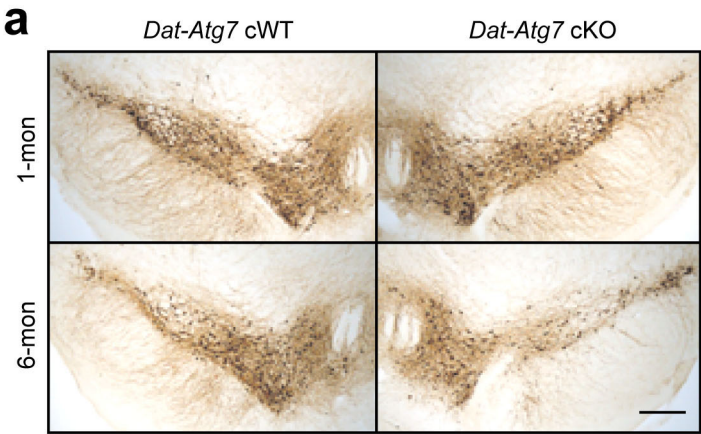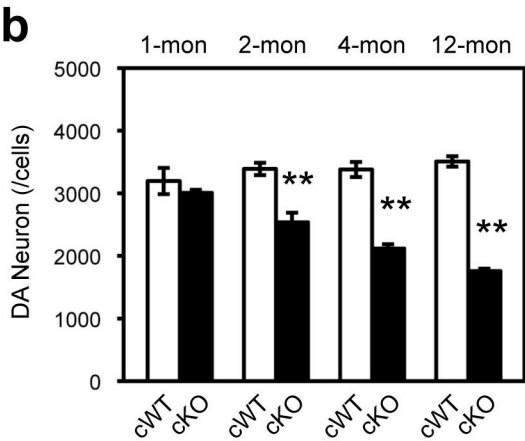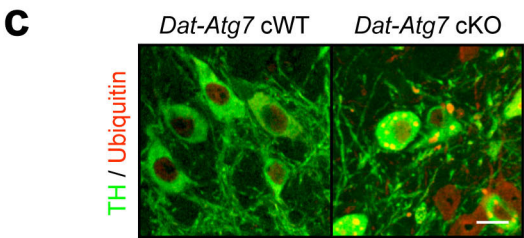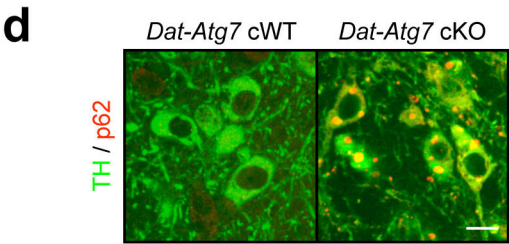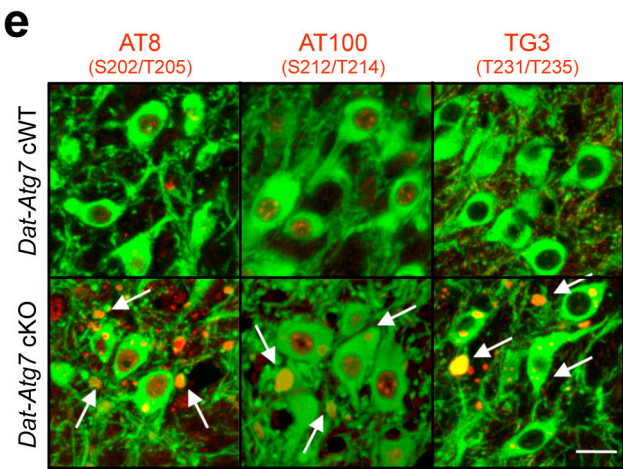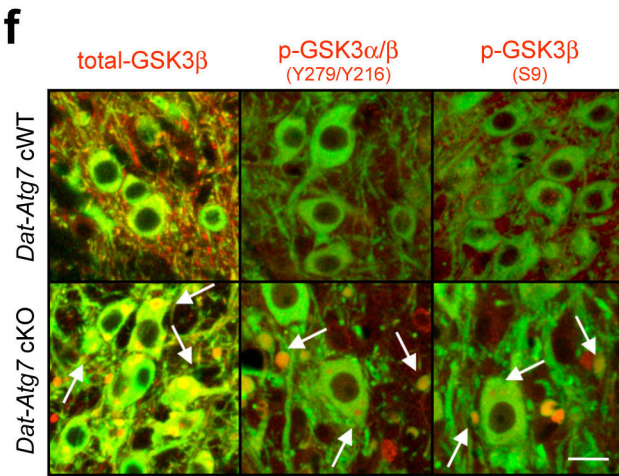

Supplement: Additional file 2 — Progressive neurodegeneration in midbrain DA neuron-specific Atg7-deficient (Dat-Atg7 cKO) mice. (a-b) Progressive loss of DA neurons in Dat-Atg7 cKO mice. a, Representative midbrain sections stained with polyclonal antibody specific for TH.Bar, 250 μm. b, Quantification of TH-positive DA neuron number as in (a). White bars, Dat-Atg7 cWT. Black bars, Dat-Atg7 cKO. n = 3 –7 for each group. **, P<0.01. (c-d) Cytoplasmic and dendritic inclusions in Dat-Atg7 cKO mice. Ubiquitin-positive (c, red) and p62/SQSTM1-positive (d, red) inclusions were present in TH-positive DA neurons (green) of 1-month-old Dat-Atg7 cKO mice, but were never seen in control Dat-Atg7 cWT mice. Bars, 10 μm. (e) Phospho-tau-positive inclusions in TH-positive DA neurons in Dat-Atg7 cKO mice. Phospho-tau specific antibodies (red), AT8, AT100, and TG3, stained inclusions (arrows) in the soma and dendrites of TH-positive DA neurons (green) in Dat-Atg7 cKO mice. AT8, tau phosphorylated at Ser202/Thr205. AT100, tau phosphorylated at Ser212/Thr214. TG3, tau phosphorylated at Thr231/Ser235. Bar, 10 μm. (f) GSK3β-positive inclusions in TH-positive DA neurons in Dat-Atg7 cKO mice. Antibodies recognizing total, activated form (Tyr279/Tyr216), and inactivated form (Ser9) of GSK3β (red), stained the inclusions (arrows) in TH-positive DA neurons (green) in Dat-Atg7 cKO mice. Bar, 10 μm. [file 1750-1326-7-48-S2.pdf]

# Additional File 3

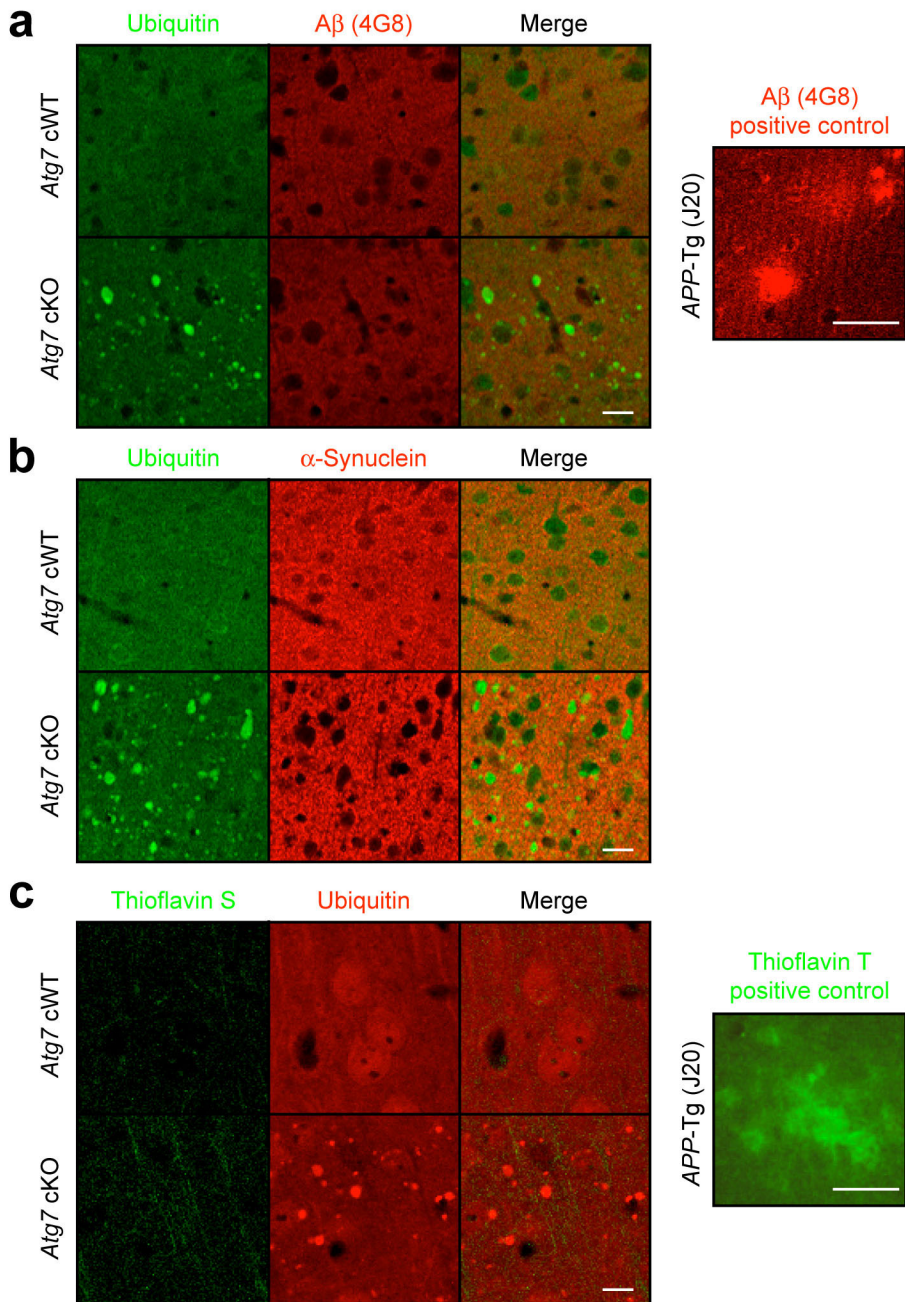

Supplement: Additional file 3 — APP/Aβ-negative, α-Synuclein-negative, and Thioflavin S-negative inclusions in CamK-Atg7 cKO mice. (a) The ubiquitin-positive inclusions (green) in 1-year-old CamK-Atg7 cKO mice did not contain mouse Aβ (red) (left). 4G8, monoclonal antibody to amino acid residues 17-24 of Aβ, was used. Ten-month-old transgenic mice bearing a mutant form of human APP (K670N/M671L/V717F, J20 line) were used as positive control for Aβ plaque staining (right). Similar negative results were obtained by 6E10, another antibody to amino acid residues 1-16 of Aβ (data not shown). Bars, 10 μm. (b) The ubiquitin-positive inclusions (green) in 1-year-old CamK-Atg7 cKO mice did not contain mouse α-Synuclein (red). Four different anti-α-synuclein antibodies were used for the double staining. None of four anti-α-synuclein antibodies could detect any positive signals (red) in ubiquitin-positive inclusions (green). Bar, 10 μm. (c) Ubiquitin-positive inclusions (red) in 1-year-old CamK-Atg7 cKO mice were negative for Thioflavin S staining (green, left). Thioflavin S stains plaques from β-amyloid and neurofibrillary tangles. Ten-month-old J20 APP transgenic mice were used as positive control for Thioflavin S staining (right). Bars, 20 μm. [file 1750-1326-7-48-S3.pdf]

# Additional File 4

**a**

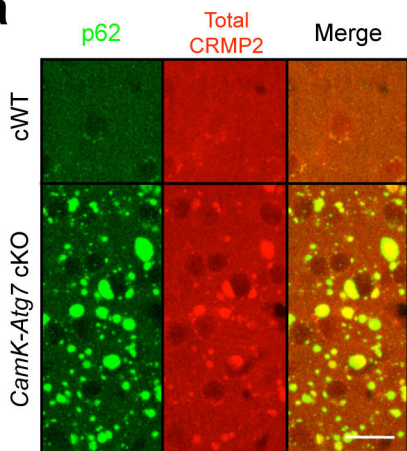

**b**

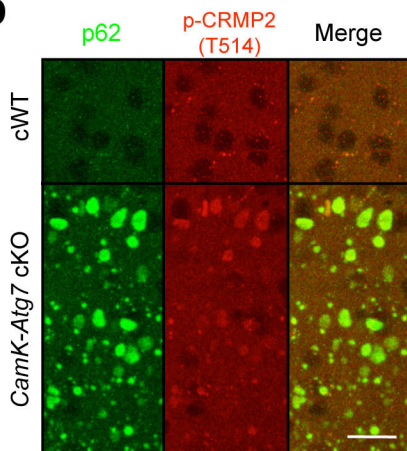

**c**

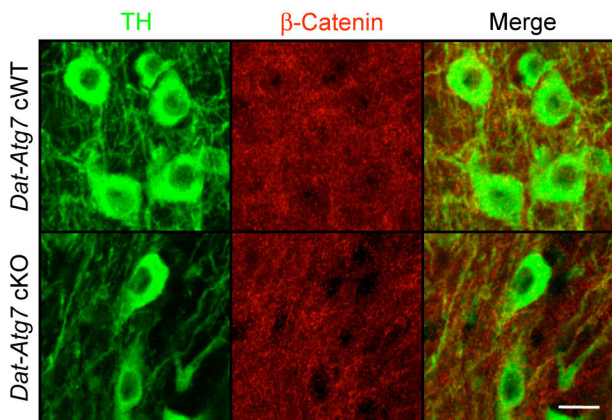

**d**

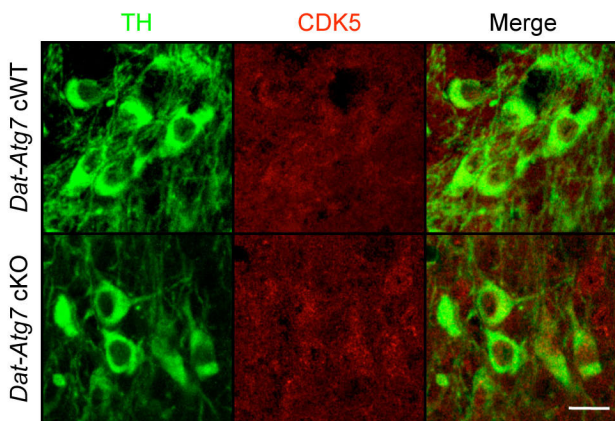

Supplement: Additional file 4 — Immunohistochemical analyses of Atg7-deficient neurons. (a-b) CRMP2-positive inclusions in cortical neurons in CamK-Atg7 cKO mice. a, An antibody recognizing total CRMP2 (red), stained p62-positive inclusions (green) in cortical neurons of CamK-Atg7 cKO mice. Bar, 10 μm. b, An antibody recognizing phosphorylated forms of CRMP2 at Thr514 residues (red), stained p62-positive inclusions (green) in cortical neurons of CamK-Atg7 cKO mice. Bar, 10 μm. (c) β-Catenin-negative inclusions in TH-positive DA neurons in Dat-Atg7 cKO mice. Antibodies recognizing β-Catenin (red) did not stain the inclusions in TH-positive DA neurons (green) in Dat-Atg7 cKO mice. Bar, 10 μm. (d) CDK5-negative inclusions in TH-positive DA neurons in Dat-Atg7 cKO mice. Antibodies recognizing CDK5 (red) did not stain the inclusions in TH-positive DA neurons (green) in Dat-Atg7 cKO mice. Bar, 10 μm. [file 1750-1326-7-48-S4.pdf]

# Additional File 5

**a**

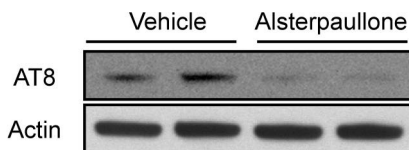

**b**

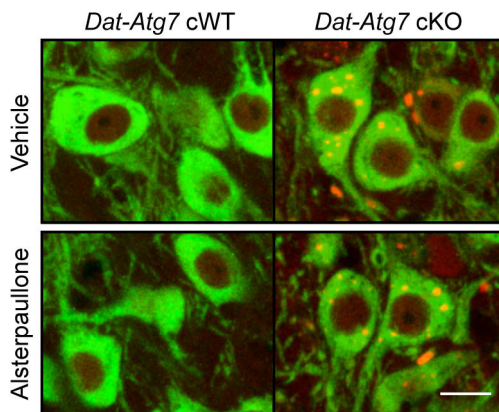

**c**

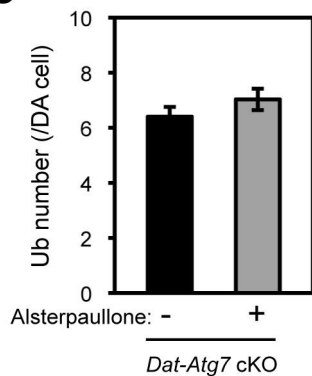

**d**

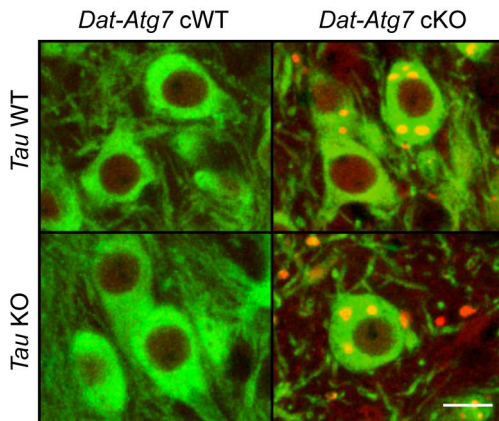

**e**

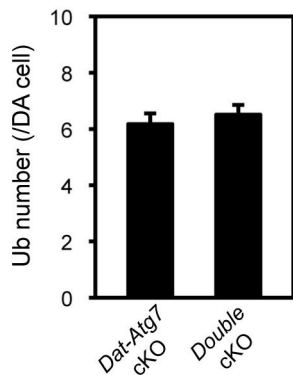

Supplement: Additional file 5 — Neuroprotection of Atg7-deficient CNS neurons in vivo. (a) Alsterpaullone can reduce phospho-tau accumulation in the context of macroautophagy inhibition. N2a cells were treated with 1 μM Alsterpaullone in the context of 100 μM chloroquine treatment for 24 h. Cells were lysed in RIPA buffer and subjected to standard Western blotting analysis. Phospho-tau levels were detected by AT8 antibody. (b-c) Ubiquitin-positive inclusion formation was unaffected by systemic injection of Alsterpaullone in the context of Dat-Atg7 cKO mice. Bar, 10 μm. c, Quantification of ubiquitin-positive inclusion number per TH-neuron in Dat-Atg7 cKO mice. No inclusions were observed in Dat-Atg7 cWT mice. n > 60 neurons per genotype. (d-e) Ubiquitin-positive inclusion formation (red) was not changed in TH-positive DA neurons (green) of Dat-Atg7/tau double cKO mice relative to Dat-Atg7 cKO mice. No ubiquitin-positive inclusions were detected in tau KO mice. Bar, 10 μm. e, Quantification of ubiquitin-positive inclusion number per TH-neuron in Dat-Atg7/tau double cKO mice. n > 60 neurons per genotype. [file 1750-1326-7-48-S5.pdf]
